# Double inferior vena cava systems during retroperitoneal surgery: Description of a systematic approach to a rare and challenging anatomic variant

**DOI:** 10.1016/j.jvscit.2021.12.003

**Published:** 2021-12-23

**Authors:** German J. Chaud, Louis Lacombe, François Dagenais

**Affiliations:** a Department of Cardiac Surgery, Quebec Heart and Lung Institute, Laval University, Quebec City, Quebec, Canada; b Department of Urology, Faculty of Medicine, Laval University, Quebec City, Quebec, Canada

**Keywords:** Extracorporeal circulation, Inferior vena cava anomalies, Left-sided inferior vena cava, Renal cell carcinoma

## Abstract

Detailed knowledge of the various venous anomalies is important to optimize the surgical approach and minimize catastrophic complications during retroperitoneal surgery. We report a rare case of an isolated left inferior vein cava (IVC) in a patient with left renal cell carcinoma with level IV IVC thrombus extension, which was successfully treated with terminal–lateral anastomoses between the left and right IVC. We also reviewed the types of duplicated IVCs and discussed the intraoperative management.

Good outcomes with treatment of renal cell carcinoma extending into the renal vein and vena cava have been shown in patients with limited disease extension.^1^ Detailed knowledge of the various venous anomalies and the amount of tumor extension are important to optimize the surgical approach. Congenital variants of the inferior vein cava (IVC) are uncommon. Most will be asymptomatic, with their diagnosis incidental during imaging.^2^ Hence, treatment is rarely required. However, owing to their close relationship to the different retroperitoneal structures, recognition of IVC congenital abnormalities is important to avoid catastrophic complications during urologic, vascular retroperitoneal surgery and endovascular procedures.^3^^,^^4^

We present a unique case of a left IVC encountered during a left radical nephrectomy for renal cell carcinoma with level IV IVC thrombus extension treated by reanastomosis to the right IVC. The patient agreed to the report of her case details and imaging studies.

## Case report

**Fig 1:**
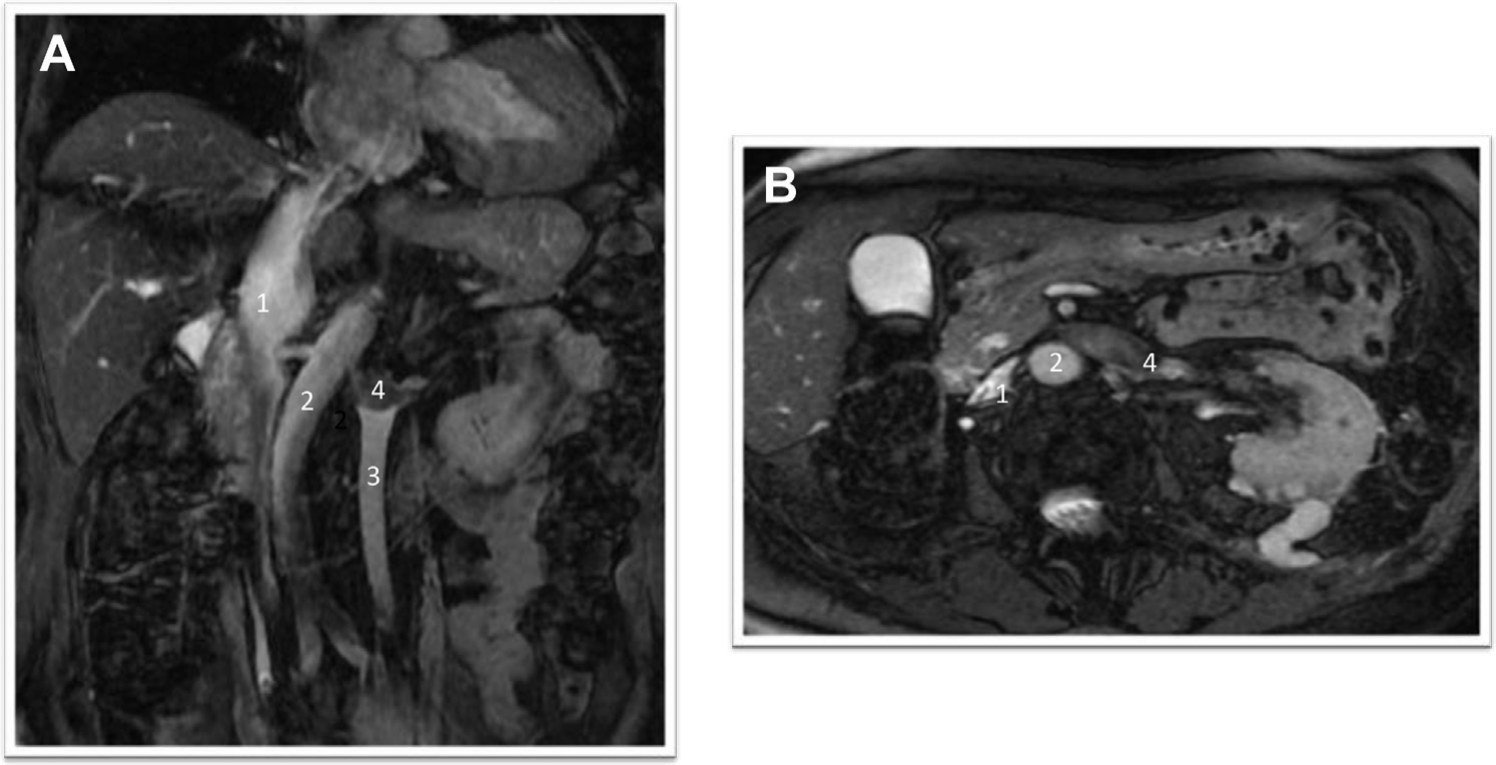
**A,** Coronal view of contrast-enhanced abdominal magnetic resonance image showing the right inferior vena cava (IVC; *1*), abdominal aorta (*2*), and left IVC (*3*) draining into a left renal vein partially obstructed by tumor (*4*). **B,** Axial view of contrast-enhanced abdominal magnetic resonance image showing right IVC (*1*) and left renal vein (*4*) with tumor over the abdominal aorta (*2*).

**Fig 2:**
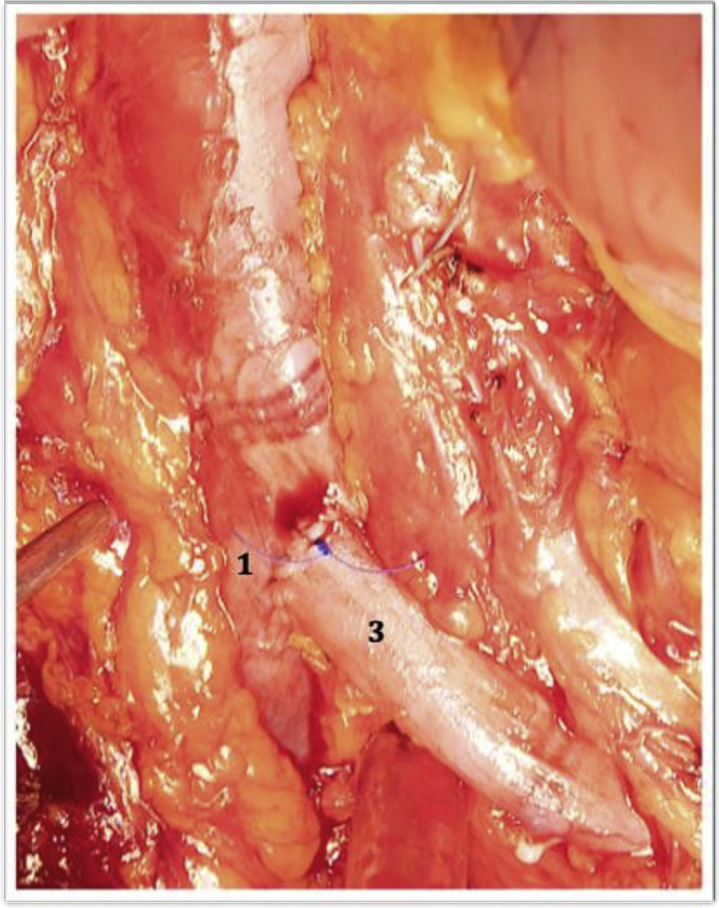
Intraoperative photograph showing the left inferior vena cava (IVC; *3*) terminal–lateral anastomosis to the right IVC (*1*).

**Fig 3:**
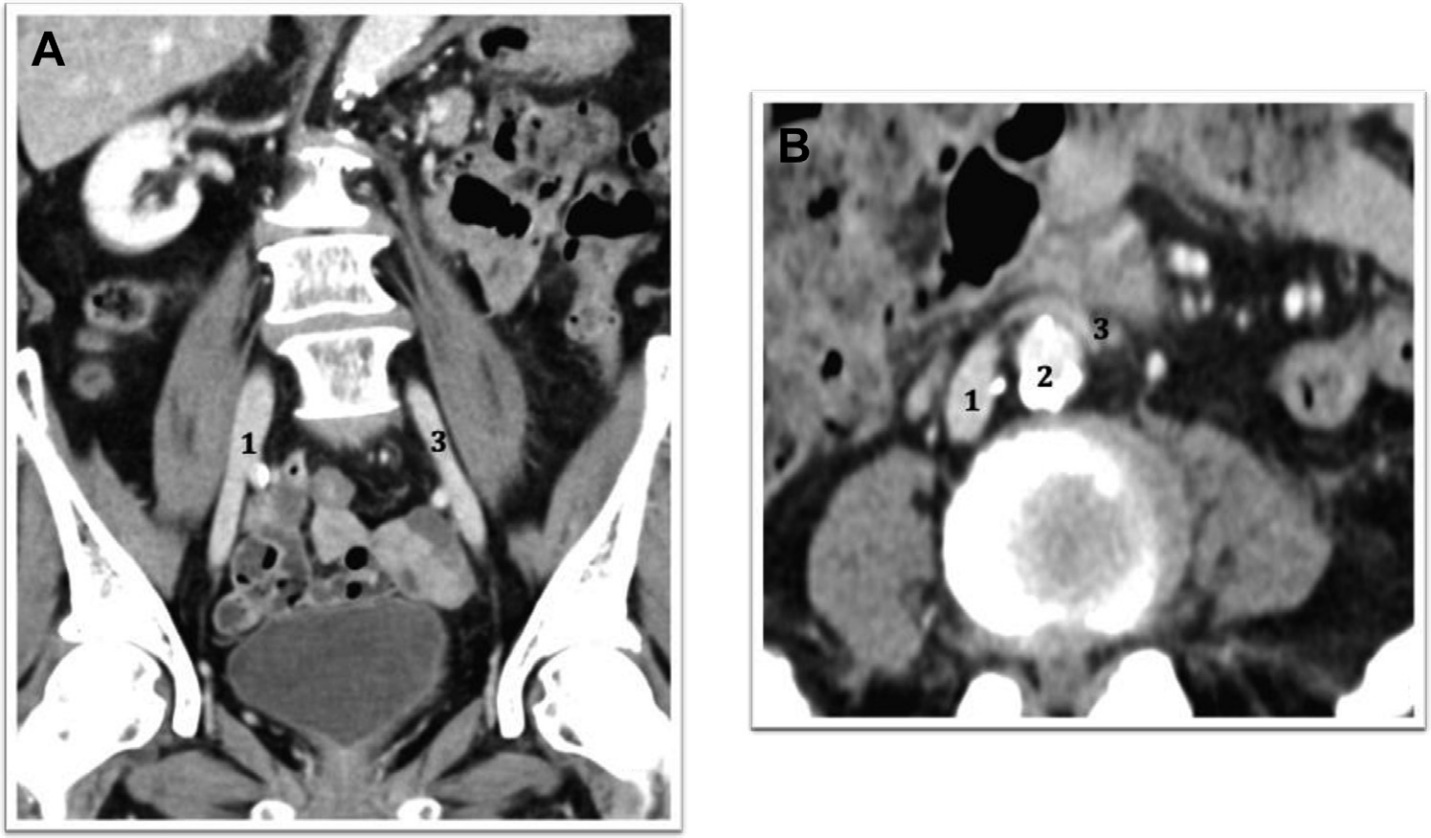
**A,** Coronal view of contrast-enhanced computed tomography scan 3 month after surgery showing a right inferior vena cava (IVC; *1*) and a patent left IVC (*3*). **B,** Axial view of contrast-enhanced computed tomography scan 3 month after surgery showing a patent left IVC (*3*) extending over the abdominal aorta (*2*) to the right IVC (*1*).

An otherwise healthy 79-year-old woman had presented with a 4 × 3-cm left kidney tumor that extended into the IVC up to the right atrium (level IV). On magnetic resonance imaging, a duplicated IVC was seen, with the left IVC draining into the partially obstructed left renal vein (Fig 1). Complementary imaging ruled out distant disease. She underwent surgery through a combined sternotomy and midline laparotomy approach. The left IVC, which was not dilated and was free of tumor, was mobilized completely to the level of the common iliac vein before extracorporeal circulation initiation. No venous collateral vessels were noted between the iliac veins. Owing to the dense adhesions surrounding the left kidney, kidney mobilization had required partial resection of the left diaphragm and splenectomy. After heparinization, the left IVC was ligated at the level of the left renal vein. Subsequently, cardiopulmonary bypass was initiated between the superior vein cava and the main pulmonary artery for venous return and arterial inflow by suturing an 8-mm Dacron graft to the innominate artery. The patient was cooled to 32°C, and the heart was arrested by clamping the ascending aorta. Owing to the innominate artery inflow, low flow unilateral selective cerebral perfusion (8 mL/kg/min) was obtained by clamping the proximal innominate artery and snaring the superior vein cava venous cannula. In bloc tumor thrombectomy was completed by opening the right atrium and the suprarenal IVC extending from the level of the left renal ostium. Primary closure of the right atrium and suprarenal IVC allowed for restoration of full cardiopulmonary bypass flow by releasing the innominate clamp after 7 minutes of low cerebral flow. During the rewarming phase, the left IVC was positioned over the abdominal aorta and anastomosed without tension in terminal–lateral fashion to the right IVC (Fig 2). Histologic examination confirmed clear cell carcinoma of the kidney with thrombus extension. At 3 months, the patient was doing well. Computed tomography scanning showed no recurrent disease and a patent left IVC to right IVC anastomosis (Fig 3).

## Discussion

The diagnosis of congenital IVC variants will most often be incidental during imaging studies for intra-abdominal pathologies.^5^ IVC development, occurring between 4 and 8 weeks of gestation, results from a complex set of anastomoses among three symmetric embryonic veins: the posterior cardinal, subcardinal, and supracardinal veins. Anomalies in the evolution and involution of these venous systems will lead to congenital IVC anomalies. More specifically, anomalies in the development of the supracardinal vein system can lead to a left-sided IVC. Persistence of both the left and the right supracardinal veins, reported in 1% to 3% of the population, results in a duplicated IVC system. In this setting, two distinct IVCs arise from each iliac vein, with the left IVC crossing over the aorta to join the right IVC at the level of the left renal vein. Less often, the left IVC will cross to the right side at a lower level than the renal vein, resulting in asymmetric IVC sizes. In contrast, regression of the right supracardinal vein and persistence of the left supracardinal vein will result in a left IVC.^6^ In this rare anomaly (prevalence, 0.2%-0.5%), the venous drainage will usually be reversed, with the gonadal and adrenal left veins draining into the left IVC and drainage of the right-sided homologous vessels joining the right renal vein.^5^ In the incomplete left IVC anomaly, the left common iliac vein will ascend as a left IVC and join the left renal vein, which, thereafter, joins the right IVC after crossing the aorta anteriorly. Rarely, the presence of an additional interiliac vein can enhance communication between both systems.^7^ In the present report, we have illustrated a unique case of an incomplete left IVC in the case of left hypernephroma with thrombus extending in the right atrium.

Congenital anomalies of the IVC can lead to three clinical issues: misdiagnosis on imaging studies, challenges in planning IVC filter insertion, and, as in the present case, technical challenges during retroperitoneal surgery.^3^^,^^7^^,^^8^ Preoperative knowledge of the presence of the abnormal venous structure will greatly facilitate management. In emergent surgeries, such as ruptured abdominal aortic aneurysms, the left vena cava can be mistaken for lymphadenopathy, possibly leading to catastrophic complications.^9^ During elective vascular surgery, the left IVC or double IVC can be mobilized by dividing the gonadal and adrenal veins, allowing for performance of clamping and replacement in a standard fashion.^10^ Occasionally, the confluence of a double IVC will require division, with subsequent terminal–terminal reanastomosis to access the aorta. In a series of nine patients who had undergone abdominal aortic surgery with IVC anomalies, including seven with a left IVC and two with a duplicated IVC,^10^ the left IVC was managed by transection and later terminal–terminal reconstruction in seven patients and through extensive IVC mobilization in two patients. Only one case of late deep vein thrombosis in the left calf was encountered.^10^

In the absence of a communicating vein between both IVC systems, a rare finding, simple ligation of a duplicated IVC is unadvisable. Ligation can be associated with lower limb stasis with edema and extensive thrombus, especially if concomitant radical lymphadenectomy is performed.^11^ However, Levack et al^9^ reported a case with ligation of a duplicated IVC without complications, arguing for the presence of abundant venous pelvic collateral vessels as compensatory channels.^3^^,^^12^ Hence ligation could be a bailout option in emergent cases to salvage hemorrhagic complications. In addition, ligation can be considered for chronic left IVC occlusion such as can occur with tumor thrombus occluding a left IVC.^13^ In such a setting, the left IVC will be dilated and the absence of left lower limb edema will suggest the presence of acceptable venous collateral circulation. In the present patient, the left IVC was not dilated, albeit not obstructed by the tumor thrombus. Reconstruction was performed by full mobilization of the incomplete left IVC with direct anastomosis to the right IVC. In the case of tension or insufficient length, interposition of an autologous tubulated vein is preferable to the use of a synthetic conduit, although excellent outcomes have been reported with Dacron and polytetrafluoroethylene conduits.^11^

## Conclusions

Anomalies of the IVC are rare but represent a challenge for surgeons treating retroperitoneal pathologies. The preoperative diagnosis remains the cornerstone to establish a surgical strategy and prevent major vascular injuries. Ligation should be limited to salvage hemorrhagic complications or in presence of a chronically occluded left IVC. Mobilization and reanastomosis of the left IVC will be possible in most cases and will ensure patent venous drainage to the left lower limb. We have reported a case of incomplete left IVC in a patient with left renal cell carcinoma, which was managed by extensive mobilization and anastomosis to the right IVC in a terminal–lateral fashion.
